# 3-[(*E*)-1-(Benzyl­oxyimino)eth­yl]-2-oxo-2*H*-chromen-7-yl acetate

**DOI:** 10.1107/S1600536810003454

**Published:** 2010-02-03

**Authors:** Hui Wang, Su-hua Xu, Zhuo Zeng, Yong-hong Zhang

**Affiliations:** a School of Chemistry and Environment, South China Normal University, Guangzhou 510006, People’s Republic of China

## Abstract

The title compound, C_20_H_17_NO_5_, was prepared by the reaction of 3-acetyl-2-oxo-2*H*-chromen-7-yl acetate with benzyl­oxy­amine. The mol­ecule adopts an *E* configuration with respect to the C=N double bond. The dihedral angles between the coumarin ring system, the phenyl ring and the C=N—O—C plane of the oxime unit are 35.83 (6), 35.8 (2) and 69.99 (15)°, respectively. In the crystal, a two-dimensional supra­molecular network is assembled through weak inter­molecular C—H⋯O hydrogen-bonding inter­actions.

## Related literature

For the pharmacological applications of Schiff base compounds derived from coumarins, see: Jolanta *et al.* (2006 ▶); Kontogiorgis *et al.* (2006 ▶); Kontogiorgis & Hadjipavlou-Litina (2004 ▶); Nofal *et al.* (2000 ▶). For their use as dyes, fluorescent agents and as chemosensors, see: Kachkovski *et al.* (2004 ▶); Turki *et al.* (2006 ▶); Li *et al.* (2009 ▶).
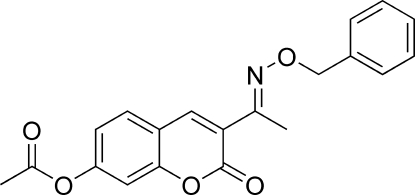


## Experimental

### 

#### Crystal data

C_20_H_17_NO_5_
*M* *_r_* = 351.35
Triclinic, 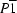

*a* = 6.3901 (9) Å
*b* = 11.2413 (16) Å
*c* = 12.9298 (18) Å
α = 102.643 (2)°
β = 96.923 (2)°
γ = 101.860 (2)°
*V* = 873.5 (2) Å^3^
*Z* = 2
Mo *K*α radiation
μ = 0.10 mm^−1^
*T* = 298 K
0.25 × 0.20 × 0.18 mm

#### Data collection

Bruker APEXII area-detector diffractometer
Absorption correction: multi-scan (*SADABS*; Bruker, 2004 ▶) *T*_min_ = 0.977, *T*_max_ = 0.983
5311 measured reflections
3792 independent reflections
2335 reflections with *I* > 2σ(*I*)
*R*_int_ = 0.025

#### Refinement

*R*[*F* ^2^ > 2σ(*F* ^2^)] = 0.051
*wR*(*F* ^2^) = 0.166
*S* = 1.09
3792 reflections
238 parameters
H-atom parameters constrained
Δρ_max_ = 0.33 e Å^−3^
Δρ_min_ = −0.23 e Å^−3^

### 

Data collection: *APEX2* (Bruker, 2004 ▶); cell refinement: *SAINT* (Bruker, 2004 ▶); data reduction: *SAINT*; program(s) used to solve structure: *SHELXS97* (Sheldrick, 2008 ▶); program(s) used to refine structure: *SHELXL97* (Sheldrick, 2008 ▶); molecular graphics: *XP* in *SHELXTL* (Sheldrick, 2008 ▶); software used to prepare material for publication: *SHELXL97*.

## Supplementary Material

Crystal structure: contains datablocks global, I. DOI: 10.1107/S1600536810003454/sj2717sup1.cif

Structure factors: contains datablocks I. DOI: 10.1107/S1600536810003454/sj2717Isup2.hkl

Additional supplementary materials:  crystallographic information; 3D view; checkCIF report

## Figures and Tables

**Table 1:** Hydrogen-bond geometry (Å, °)

*D*—H⋯*A*	*D*—H	H⋯*A*	*D*⋯*A*	*D*—H⋯*A*
C6—H6⋯O5^i^	0.93	2.51	3.393 (3)	159
C18—H18⋯O5^ii^	0.93	2.67	3.552 (3)	160
C8—H8⋯O1^iii^	0.93	2.65	3.397 (2)	138

Symmetry codes: (i) 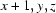; (ii) 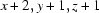; (iii) 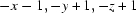.

**Fig. 1.**
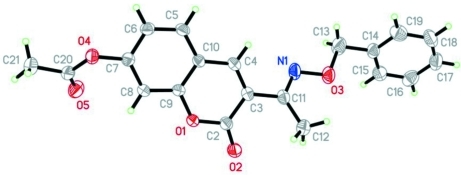
The molecular structure of the title compound showing the atom numbering scheme. Displacement ellipsoids are drawn at the 50% probability level.

**Fig. 2.**
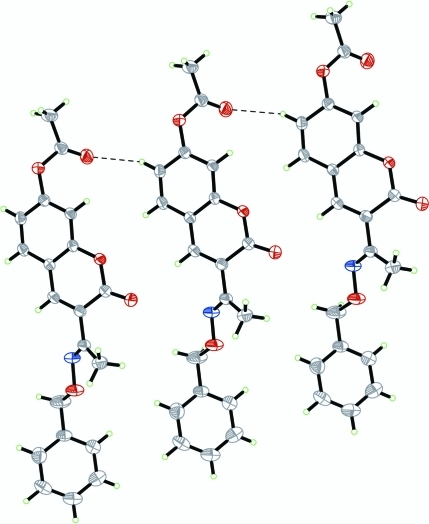
Part of the crystal structure of the title compound showing weak C—H···O hydrogen bonds as dashed lines.

**Fig. 3.**
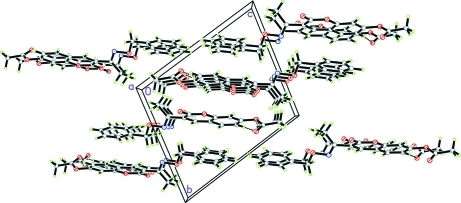
Crystal packing in the title compound.

## supplementary crystallographic information

### Comment

Coumarin-derived Schiff bases have attracted much attention due to their potential pharmacological applications as antitumor (Jolanta *et al.*, 2006), anti-inflammatory (Kontogiorgis & Hadjipavlou-Litina 2004), antibacterial (Nofal *et al.*, 2000) and antifungal agents (Kontogiorgis *et al.*, 2006). In addition, they can also be used as dyes (Kachkovski *et al.*, 2004), fluorescent agents (Turki *et al.*, 2006) and as colorimetric chemosensors (Li *et al.*, 2009). In our study of bioactive compounds, a series of coumarin-derived Schiff bases have been synthesized. Herein, we report the crystal structure of the title compound, Fig. 1, obtained by the reaction of 3-acetyl-2-oxo-2*H*-chromen-7-yl acetate with benzyloxy-amine.

The title molecule is composed of a coumarin core with acetoxyl and benzyloxyiminoethyl substituents. The dihedral angles between the coumarin ring system, the phenyl ring and the C=N—O—C plane of the oxime unit are 35.83 (6)°, 35.8 (2) ° and 69.99 (15) °, respectively. The benzyloxyiminoethyl and coumarin systems are located on opposite sides of the C=N double bond plane, therefor the molecule presents an E configuration. In the crystal structure, intermolecular C6—H6···O5 hydrogen bonding interactions (Table 1) assemble a two-dimensional supramolecular layer as shown in Fig. 2. Additional weak C8—H8···O1 and C18—H18···O5 contacts are also observed.  The overall crystal packing is shown in Fig. 3.

### Experimental

A solution of benzyloxy-amine hydrochloride (2 mmol) in ethanol (10 ml) was added to a solution of 3-acetyl-2-oxo-2*H*-chromen-7-yl acetate (1 mmol) in ethanol (10 ml) at room temperature, the solution pH was then maintained at a value of 7  by the addition of sodium hydroxide. The reaction mixture was refluxed for 5 h at 353 K (monitored by TLC). After completion of the reaction, the reaction solution was purified by column chromatography (ethyl acetate: petroleum ether = 2:3). The eluate was evaporated to give the title compound (286 mg, yield: 81.5%). Single crystals suitable for X-ray analysis were obtained by recrystallization from DMSO, m.p. 426 K. ESI-MS (m/*z*): 352 (*M*+1); Analysis calculated for C_20_H_17_NO_5_: C 68.37%, H 4.88%, N 3.99%; Found: C 68.54%, H 4.32%, N 3.78%.

### Refinement

All H-atoms were positioned geometrically and refined using a riding model with d(C—H) = 0.93 Å, *U*_iso_=1.2*U*_eq_ (C) for aromatic 0.97 Å, *U*_iso_ = 1.2*U*_eq_ (C) for CH_2_ and 0.96 Å, *U*_iso_ = 1.5*U*_eq_ (C) for CH_3_ atoms.

### Crystal data

**Table:**

C_20_H_17_NO_5_	*Z* = 2
*M**_r_* = 351.35	*F*(000) = 368
Triclinic, *P*1	*D*_x_ = 1.336 Mg m^−^^3^
Hall symbol: -P 1	Mo *K*α radiation, λ = 0.71073 Å
*a* = 6.3901 (9) Å	Cell parameters from 1332 reflections
*b* = 11.2413 (16) Å	θ = 2.8–24.5°
*c* = 12.9298 (18) Å	µ = 0.10 mm^−^^1^
α = 102.643 (2)°	*T* = 298 K
β = 96.923 (2)°	Block, colorless
γ = 101.860 (2)°	0.25 × 0.20 × 0.18 mm
*V* = 873.5 (2) Å^3^	

### Data collection

**Table:**

Bruker APEXII area-detector diffractometer	3792 independent reflections
Radiation source: fine-focus sealed tube	2335 reflections with *I* > 2σ(*I*)
graphite	*R*_int_ = 0.025
φ and ω scans	θ_max_ = 27.3°, θ_min_ = 2.2°
Absorption correction: multi-scan (*SADABS*; Bruker, 2004)	*h* = −8→6
*T*_min_ = 0.977, *T*_max_ = 0.983	*k* = −12→14
5311 measured reflections	*l* = −16→16

### Refinement

**Table:**

Refinement on *F*^2^	Secondary atom site location: difference Fourier map
Least-squares matrix: full	Hydrogen site location: inferred from neighbouring sites
*R*[*F*^2^ > 2σ(*F*^2^)] = 0.051	H-atom parameters constrained
*wR*(*F*^2^) = 0.166	*w* = 1/[σ^2^(*F*_o_^2^) + (0.076*P*)^2^ + 0.0611*P*] where *P* = (*F*_o_^2^ + 2*F*_c_^2^)/3
*S* = 1.09	(Δ/σ)_max_ < 0.001
3792 reflections	Δρ_max_ = 0.33 e Å^−^^3^
238 parameters	Δρ_min_ = −0.23 e Å^−^^3^
0 restraints	Extinction correction: *SHELXL97* (Sheldrick, 2008), Fc^*^=kFc[1+0.001xFc^2^λ^3^/sin(2θ)]^-1/4^
Primary atom site location: structure-invariant direct methods	Extinction coefficient: 0.010 (4)

### Special details

**Table:**

Experimental. FT—IR (KBr): 1763, 1717, 1615, 1426, 1365, 1200, 1030 cm^-1^; ^1^H NMR δ (400 Hz, DMSO, TMS): 8.08 (s, 1H), 7.83 (d, J = 8.4 Hz, 1H), 7.36–7.28 (m, 5H) 7.26 (d, J = 2.0 Hz, 1H), 7.14 (dd, J = 2.0, 8.4 Hz, 1H), 5.17 (s, 2H), 2.27 (s, 3H), 2.11 (s, 3H).
Geometry. All e.s.d.'s (except the e.s.d. in the dihedral angle between two l.s. planes) are estimated using the full covariance matrix. The cell e.s.d.'s are taken into account individually in the estimation of e.s.d.'s in distances, angles and torsion angles; correlations between e.s.d.'s in cell parameters are only used when they are defined by crystal symmetry. An approximate (isotropic) treatment of cell e.s.d.'s is used for estimating e.s.d.'s involving l.s. planes.
Refinement. Refinement of *F*^2^ against ALL reflections. The weighted *R*-factor *wR* and goodness of fit *S* are based on *F*^2^, conventional *R*-factors *R* are based on *F*, with *F* set to zero for negative *F*^2^. The threshold expression of *F*^2^ > σ(*F*^2^) is used only for calculating *R*-factors(gt) *etc*. and is not relevant to the choice of reflections for refinement. *R*-factors based on *F*^2^ are statistically about twice as large as those based on *F*, and *R*- factors based on ALL data will be even larger.

### Fractional atomic coordinates and isotropic or equivalent isotropic displacement parameters (Å^2^)

**Table:**

	*x*	*y*	*z*	*U*_iso_*/*U*_eq_	
O1	−0.33761 (19)	0.48915 (12)	0.62680 (10)	0.0488 (4)	
C2	−0.2517 (3)	0.56481 (18)	0.72817 (15)	0.0499 (5)	
O2	−0.3700 (2)	0.62098 (15)	0.77276 (12)	0.0710 (5)	
C3	−0.0274 (3)	0.56859 (17)	0.77165 (15)	0.0472 (5)	
C4	0.0814 (3)	0.49479 (17)	0.71383 (15)	0.0478 (5)	
H4	0.2234	0.4970	0.7421	0.057*	
C5	0.0893 (3)	0.33485 (18)	0.54693 (16)	0.0504 (5)	
H5	0.2301	0.3320	0.5725	0.060*	
C6	−0.0133 (3)	0.26229 (17)	0.44758 (16)	0.0503 (5)	
H6	0.0568	0.2097	0.4062	0.060*	
C7	−0.2229 (3)	0.26729 (17)	0.40849 (15)	0.0448 (4)	
O4	−0.3080 (2)	0.19654 (12)	0.30368 (10)	0.0542 (4)	
C20	−0.5180 (4)	0.12835 (19)	0.27716 (18)	0.0591 (6)	
O5	−0.6377 (3)	0.12328 (15)	0.34060 (14)	0.0791 (5)	
C21	−0.5679 (4)	0.0639 (2)	0.1608 (2)	0.0821 (8)	
H21C	−0.7167	0.0173	0.1424	0.123*	
H21A	−0.4749	0.0076	0.1455	0.123*	
H21B	−0.5445	0.1251	0.1194	0.123*	
C8	−0.3309 (3)	0.34359 (17)	0.46779 (14)	0.0450 (4)	
H8	−0.4706	0.3471	0.4410	0.054*	
C9	−0.2257 (3)	0.41476 (16)	0.56822 (14)	0.0415 (4)	
C10	−0.0149 (3)	0.41373 (16)	0.61081 (14)	0.0427 (4)	
C11	0.0738 (3)	0.65509 (19)	0.87771 (16)	0.0539 (5)	
C12	0.0328 (4)	0.7832 (2)	0.90913 (19)	0.0729 (7)	
H12C	0.1607	0.8404	0.9536	0.109*	
H12B	−0.0857	0.7804	0.9484	0.109*	
H12A	−0.0030	0.8111	0.8456	0.109*	
N1	0.2041 (3)	0.61121 (16)	0.93402 (13)	0.0644 (5)	
O3	0.3083 (3)	0.70165 (14)	1.03000 (11)	0.0744 (5)	
C13	0.4579 (5)	0.6475 (3)	1.0870 (2)	0.0940 (9)	
H13B	0.5690	0.6295	1.0452	0.113*	
H13A	0.3816	0.5700	1.1006	0.113*	
C14	0.5568 (4)	0.74251 (19)	1.19015 (17)	0.0609 (6)	
C15	0.4399 (4)	0.7731 (2)	1.26843 (19)	0.0677 (6)	
H15	0.2937	0.7326	1.2573	0.081*	
C16	0.5268 (5)	0.8596 (2)	1.3618 (2)	0.0812 (8)	
H16	0.4415	0.8772	1.4139	0.097*	
C17	0.7365 (5)	0.9205 (2)	1.3795 (2)	0.0820 (8)	
H17	0.7944	0.9811	1.4437	0.098*	
C18	0.8661 (4)	0.8957 (3)	1.3058 (2)	0.0842 (8)	
H18	1.0115	0.9384	1.3194	0.101*	
C19	0.7778 (4)	0.8050 (3)	1.2092 (2)	0.0817 (7)	
H19	0.8644	0.7862	1.1579	0.098*	

### Atomic displacement parameters (Å^2^)

**Table:**

	*U*^11^	*U*^22^	*U*^33^	*U*^12^	*U*^13^	*U*^23^
O1	0.0384 (7)	0.0550 (8)	0.0484 (8)	0.0149 (6)	0.0013 (6)	0.0035 (6)
C2	0.0446 (11)	0.0558 (11)	0.0473 (11)	0.0116 (9)	0.0061 (9)	0.0102 (9)
O2	0.0508 (9)	0.0869 (11)	0.0646 (10)	0.0236 (8)	0.0070 (7)	−0.0079 (8)
C3	0.0426 (10)	0.0514 (11)	0.0448 (10)	0.0066 (8)	0.0044 (8)	0.0123 (8)
C4	0.0359 (10)	0.0543 (11)	0.0510 (11)	0.0074 (8)	0.0004 (8)	0.0153 (9)
C5	0.0354 (10)	0.0531 (11)	0.0627 (12)	0.0126 (8)	0.0064 (9)	0.0138 (9)
C6	0.0430 (11)	0.0463 (10)	0.0618 (12)	0.0125 (8)	0.0130 (9)	0.0103 (9)
C7	0.0427 (10)	0.0419 (9)	0.0480 (11)	0.0059 (8)	0.0076 (8)	0.0118 (8)
O4	0.0472 (8)	0.0580 (8)	0.0494 (8)	0.0078 (6)	0.0067 (6)	0.0023 (6)
C20	0.0505 (13)	0.0480 (11)	0.0688 (14)	0.0118 (9)	0.0011 (11)	−0.0009 (10)
O5	0.0550 (10)	0.0705 (10)	0.0932 (12)	−0.0003 (8)	0.0205 (9)	−0.0079 (9)
C21	0.0753 (16)	0.0762 (16)	0.0731 (16)	0.0155 (13)	−0.0072 (13)	−0.0128 (13)
C8	0.0374 (10)	0.0488 (10)	0.0481 (11)	0.0125 (8)	0.0029 (8)	0.0109 (8)
C9	0.0377 (10)	0.0428 (9)	0.0464 (10)	0.0126 (7)	0.0081 (8)	0.0128 (8)
C10	0.0364 (10)	0.0432 (9)	0.0472 (10)	0.0058 (7)	0.0054 (8)	0.0134 (8)
C11	0.0495 (12)	0.0610 (12)	0.0456 (11)	0.0079 (9)	0.0042 (9)	0.0091 (9)
C12	0.0735 (16)	0.0703 (15)	0.0628 (14)	0.0169 (12)	0.0027 (12)	−0.0030 (11)
N1	0.0720 (12)	0.0609 (11)	0.0453 (10)	0.0069 (9)	−0.0095 (9)	0.0015 (8)
O3	0.0890 (12)	0.0662 (10)	0.0523 (9)	0.0159 (8)	−0.0181 (8)	0.0014 (7)
C13	0.126 (2)	0.0769 (17)	0.0661 (16)	0.0340 (16)	−0.0280 (15)	0.0053 (13)
C14	0.0722 (15)	0.0542 (12)	0.0487 (12)	0.0134 (11)	−0.0092 (11)	0.0092 (9)
C15	0.0686 (15)	0.0641 (14)	0.0688 (15)	0.0136 (11)	0.0064 (12)	0.0186 (12)
C16	0.109 (2)	0.0721 (16)	0.0676 (16)	0.0310 (16)	0.0149 (15)	0.0201 (13)
C17	0.099 (2)	0.0624 (15)	0.0700 (17)	0.0142 (15)	−0.0147 (16)	0.0070 (13)
C18	0.0612 (16)	0.0781 (17)	0.098 (2)	−0.0013 (13)	−0.0165 (15)	0.0231 (15)
C19	0.0776 (18)	0.0987 (19)	0.0770 (17)	0.0287 (15)	0.0165 (14)	0.0307 (15)

### Geometric parameters (Å, °)

**Table:**

O1—C9	1.372 (2)	C9—C10	1.396 (2)
O1—C2	1.375 (2)	C11—N1	1.283 (3)
C2—O2	1.202 (2)	C11—C12	1.494 (3)
C2—C3	1.464 (3)	C12—H12C	0.9600
C3—C4	1.350 (3)	C12—H12B	0.9600
C3—C11	1.479 (3)	C12—H12A	0.9600
C4—C10	1.423 (2)	N1—O3	1.410 (2)
C4—H4	0.9300	O3—C13	1.447 (3)
C5—C6	1.365 (3)	C13—C14	1.489 (3)
C5—C10	1.403 (3)	C13—H13B	0.9700
C5—H5	0.9300	C13—H13A	0.9700
C6—C7	1.390 (3)	C14—C15	1.355 (3)
C6—H6	0.9300	C14—C19	1.408 (3)
C7—C8	1.372 (3)	C15—C16	1.347 (3)
C7—O4	1.392 (2)	C15—H15	0.9300
O4—C20	1.363 (2)	C16—C17	1.340 (4)
C20—O5	1.190 (2)	C16—H16	0.9300
C20—C21	1.483 (3)	C17—C18	1.356 (4)
C21—H21C	0.9600	C17—H17	0.9300
C21—H21A	0.9600	C18—C19	1.398 (4)
C21—H21B	0.9600	C18—H18	0.9300
C8—C9	1.375 (2)	C19—H19	0.9300
C8—H8	0.9300		
			
C9—O1—C2	122.88 (14)	C5—C10—C4	124.61 (17)
O2—C2—O1	116.29 (17)	N1—C11—C3	113.44 (18)
O2—C2—C3	126.37 (18)	N1—C11—C12	125.35 (19)
O1—C2—C3	117.34 (17)	C3—C11—C12	121.10 (18)
C4—C3—C2	119.42 (17)	C11—C12—H12C	109.5
C4—C3—C11	122.05 (18)	C11—C12—H12B	109.5
C2—C3—C11	118.53 (17)	H12C—C12—H12B	109.5
C3—C4—C10	121.90 (17)	C11—C12—H12A	109.5
C3—C4—H4	119.0	H12C—C12—H12A	109.5
C10—C4—H4	119.0	H12B—C12—H12A	109.5
C6—C5—C10	120.80 (17)	C11—N1—O3	110.70 (17)
C6—C5—H5	119.6	N1—O3—C13	107.54 (16)
C10—C5—H5	119.6	O3—C13—C14	106.20 (19)
C5—C6—C7	119.80 (18)	O3—C13—H13B	110.5
C5—C6—H6	120.1	C14—C13—H13B	110.5
C7—C6—H6	120.1	O3—C13—H13A	110.5
C8—C7—C6	121.47 (17)	C14—C13—H13A	110.5
C8—C7—O4	122.92 (16)	H13B—C13—H13A	108.7
C6—C7—O4	115.48 (16)	C15—C14—C19	117.5 (2)
C20—O4—C7	120.76 (15)	C15—C14—C13	122.1 (2)
O5—C20—O4	123.2 (2)	C19—C14—C13	120.4 (2)
O5—C20—C21	127.0 (2)	C16—C15—C14	122.7 (2)
O4—C20—C21	109.8 (2)	C16—C15—H15	118.7
C20—C21—H21C	109.5	C14—C15—H15	118.7
C20—C21—H21A	109.5	C17—C16—C15	119.9 (3)
H21C—C21—H21A	109.5	C17—C16—H16	120.0
C20—C21—H21B	109.5	C15—C16—H16	120.0
H21C—C21—H21B	109.5	C16—C17—C18	121.5 (3)
H21A—C21—H21B	109.5	C16—C17—H17	119.3
C7—C8—C9	117.84 (17)	C18—C17—H17	119.3
C7—C8—H8	121.1	C17—C18—C19	119.0 (3)
C9—C8—H8	121.1	C17—C18—H18	120.5
O1—C9—C8	116.92 (15)	C19—C18—H18	120.5
O1—C9—C10	120.20 (16)	C18—C19—C14	119.4 (2)
C8—C9—C10	122.89 (16)	C18—C19—H19	120.3
C9—C10—C5	117.19 (17)	C14—C19—H19	120.3
C9—C10—C4	118.17 (16)		
			
C9—O1—C2—O2	−176.76 (17)	C8—C9—C10—C4	177.57 (16)
C9—O1—C2—C3	2.8 (3)	C6—C5—C10—C9	−0.2 (3)
O2—C2—C3—C4	176.5 (2)	C6—C5—C10—C4	−178.29 (17)
O1—C2—C3—C4	−3.0 (3)	C3—C4—C10—C9	2.0 (3)
O2—C2—C3—C11	−4.3 (3)	C3—C4—C10—C5	−179.96 (18)
O1—C2—C3—C11	176.28 (16)	C4—C3—C11—N1	−36.2 (3)
C2—C3—C4—C10	0.6 (3)	C2—C3—C11—N1	144.62 (19)
C11—C3—C4—C10	−178.60 (16)	C4—C3—C11—C12	140.3 (2)
C10—C5—C6—C7	0.7 (3)	C2—C3—C11—C12	−38.9 (3)
C5—C6—C7—C8	−0.3 (3)	C3—C11—N1—O3	175.21 (16)
C5—C6—C7—O4	175.76 (16)	C12—C11—N1—O3	−1.1 (3)
C8—C7—O4—C20	−45.7 (2)	C11—N1—O3—C13	−177.84 (19)
C6—C7—O4—C20	138.31 (18)	N1—O3—C13—C14	−177.2 (2)
C7—O4—C20—O5	−1.4 (3)	O3—C13—C14—C15	67.9 (3)
C7—O4—C20—C21	179.04 (17)	O3—C13—C14—C19	−111.6 (3)
C6—C7—C8—C9	−0.6 (3)	C19—C14—C15—C16	0.1 (3)
O4—C7—C8—C9	−176.27 (15)	C13—C14—C15—C16	−179.4 (2)
C2—O1—C9—C8	179.99 (16)	C14—C15—C16—C17	0.8 (4)
C2—O1—C9—C10	−0.1 (3)	C15—C16—C17—C18	−1.0 (4)
C7—C8—C9—O1	−179.15 (15)	C16—C17—C18—C19	0.4 (4)
C7—C8—C9—C10	1.0 (3)	C17—C18—C19—C14	0.6 (4)
O1—C9—C10—C5	179.54 (15)	C15—C14—C19—C18	−0.8 (3)
C8—C9—C10—C5	−0.6 (3)	C13—C14—C19—C18	178.8 (2)
O1—C9—C10—C4	−2.3 (2)		

### Hydrogen-bond geometry (Å, °)

**Table:**

*D*—H···*A*	*D*—H	H···*A*	*D*···*A*	*D*—H···*A*
C6—H6···O5^i^	0.93	2.51	3.393 (3)	159
C18—H18···O5^ii^	0.93	2.67	3.552 (3)	160
C8—H8···O1^iii^	0.93	2.65	3.397 (2)	138

Symmetry codes: (i) *x*+1, *y*, *z*; (ii) *x*+2, *y*+1, *z*+1; (iii) −*x*−1, −*y*+1, −*z*+1.
